# Design of InnoPrimers-Duplex Real-Time PCR for Detection and Treatment Response Prediction of EBV-Associated Nasopharyngeal Carcinoma Circulating Genetic Biomarker

**DOI:** 10.3390/diagnostics11101761

**Published:** 2021-09-25

**Authors:** Mai Abdel Haleem Abusalah, Siti Asma Binti Hassan, Norhafiza Mat Lazim, Baharudin Abdullah, Wan Fatihah Binti Wan Sohaimi, Azlan Husin, Kueh Yee Cheng, Chan Yean Yean

**Affiliations:** 1 Department of Medical Microbiology and Parasitology, School of Medical Sciences, Health Campus, Universiti Sains Malaysia, Kubang Kerian, Kota Bharu 16150, Kelantan, Malaysia; maiabdelhaleem@student.usm.my (M.A.H.A.); sitiasmakb@usm.my (S.A.B.H.); 2 Hospital USM, Health Campus, Universiti Sains Malaysia, Kubang Kerian, Kota Bharu 16150, Kelantan, Malaysia; norhafiza@usm.my (N.M.L.); baharudin@usm.my (B.A.); fatihahkk@usm.my (W.F.B.W.S.); azlanh@usm.my (A.H.); yckueh@usm.my (K.Y.C.); 3 Department of Otorhinolaryngology-Head and Neck Surgery, School of Medical Sciences, Health Campus, Universiti Sains Malaysia, Kubang Kerian, Kota Bharu 16150, Kelantan, Malaysia; 4 Department of Nuclear Medicine, Radiotherapy and Oncology, School of Medical Sciences, Health Campus, Universiti Sains Malaysia, Kubang Kerian, Kota Bharu 16150, Kelantan, Malaysia; 5 Department of Internal Medicine, School of Medical Sciences, Health Campus, Universiti Sains Malaysia, Kubang Kerian, Kota Bharu 16150, Kelantan, Malaysia; 6 Biostatistics and Research Methodology Unit, Health Campus, Universiti Sains Malaysia, Kubang Kerian, Kota Bharu 16150, Kelantan, Malaysia

**Keywords:** Epstein-Barr virus, latent membrane protein 1, 30 bp deletion NPC genetic biomarker, real-time PCR, nasopharyngeal carcinoma

## Abstract

Nasopharyngeal carcinoma (NPC) is an epithelial tumor with high prevalence in southern China and Southeast Asia. NPC is well associated with the Epstein-Barr virus (EBV) latent membrane protein 1 (LMP1) 30 bp deletion by having its vital role in increased tumorigenicity and decreased immune recognition of EBV-related tumors. This study developed an InnoPrimers-duplex qPCR for detection of NPC blood circulating LMP1 30 bp deletion genetic biomarker for early diagnosis and treatment response prediction of NPC patients. The analytical and diagnostic evaluation and treatment response prediction were conducted using NPC patients’ whole blood (WB) and tissue samples and non-NPC cancer patients and healthy individuals’ WB samples. The assay was able to detect as low as 20 ag DNA per reaction (equivalent to 173 copies) with high specificity against broad reference microorganisms and archive NPC biopsy tissue and FNA samples. The diagnostic sensitivity and specificity were 83.3% and 100%, respectively. The 30 bp deletion genetic biomarker was found to be a good prognostic biomarker associated with overall clinical outcome of NPC WHO type III patients. This sensitive and specific assay can help clinicians in early diagnosis and treatment response prediction of NPC patients, which will enhance treatment outcome and lead to better life-saving.

## 1. Introduction

Nasopharyngeal carcinoma (NPC) is a non-lymphomatous squamous cell carcinoma that develops in the epithelial cells layer that line the surface of the nasopharynx [1,2]. NPC is a distinct form of head and neck cancer in terms of its etiology, clinical presentation, pathology, geographical and racial distribution and response to treatment [2,3]. Globally, NPC is an uncommon malignancy with an occurrence rate of usually <1 per 100,000 person-years, but it was reported with high incidence rate in certain regions such as southern China (e.g., Cantonese), Southeast Asia (e.g., Sarawak Bidayuh), North Africa and the Arctic (e.g., Inuit, Alaska native). Approximately 71% of new NPC cases are registered in East and Southeast Asia, and 29% are diagnosed in South and Central Asia and North and East Africa [4]. There are three major etiological factors for NPC, including genetic susceptibility, environmental and dietary factors and Epstein–Barr virus (EBV) infection [5,6,7]. The strong association between NPC and EBV infection was reported in non-keratinizing carcinoma (NKC), which is divided into non-keratinizing differentiated carcinoma (NKDC) (type II) and non-keratinizing undifferentiated carcinoma (NKUC) (type III) [8,9].

The presence of a broad spectrum of clinical symptoms is often confusing in early stages until patients have advanced stages, so early diagnosis of NPC is crucial in treatment effectiveness and prognosis of NPC patients [2,10]. Clinically, at an early stage, NPC is asymptomatic or has non-specific symptoms; more than 80% of NPC patients are first diagnosed at a late stage (III or IV), frequently with metastasis of the cervical lymph node, leading to decreased survival [11,12]. However, the patient history, physical examination and imaging (CT and MRI) are critical in establishing the correct diagnosis; taking biopsy samples using nasoendoscopy is considered the gold standard, definitive and confirmatory approach for NPC diagnosis and prognosis [13,14,15]. Moreover, additional diagnostic tests such as diagnostic imaging assessment and serology test are needed in establishing the correct diagnosis [10].

A wide range of assays was utilized in the diagnosis of EBV. In the last 20 years, several serological tests have been used as a screening tool for NPC [16,17]. The available serological assays were reported with low sensitivity and specificity rates in detecting EBV DNA and in diagnosing NPC patients, especially at early stages; hence, the development of more sensitive and specific assay for early diagnosis of NPC patients was required [18]. In addition, other diagnostic tools to detect EBV-associated NPC such as and molecular sequencing detection, immunohistochemistry and immunohistology methods been used, but these assays are invasive and require at least 2 weeks to obtain the result [16,17]. Although various methods have been advocated in assisting the diagnosis of EBV-associated NPC, unfortunately, the sensitivity and specificity of these tests are known to be insufficient [16,17]. In contrast, serum or plasma level of EBV viral load was reported to be helpful in monitoring of treatment effectiveness and predicts recurrence, prognosis and diagnosis of NPC patients [19,20,21,22]. Currently in the clinical evaluation of EBV-associated tumor, the molecular determination of EBV DNA, RNA and EBV viral load is widely used [23,24].

Various studies detected the presences of EBV viral load in NPC patients by targeting different EBV latent genes such as LMP2, EBNA1 and Bam HI-W region of the EBV genome. However, The Bam HI-W region was reported as an excellent target for highly sensitive PCR because it was found in multiple copies (7–11 copies per genome) [25,26], the variability in Bam H1-W copy numbers may add an imprecision in EBV quantification, and it may cause inaccurate viral load detection [25]. Other literatures reported that EBNA1 is the only latent protein expressed in all EBV-associated carcinomas [27,28,29]. However, EBNA1 does not have a significant role in the transformation of vitro B cells and the pathogenesis of NPC as reported by other studies [30,31]. In contrast, LMP1 is considered the primary oncogene of EBV that responsible for the malignant phenotype in NPC, and the expression of LMP1 was reported to be higher than LMP2 in NPC. LMP1 played an important role in treatment resistance, decreased survival and promote metastasis [32]. In addition, the LMP1 30 bp deletion was reported to increase the tumorigenesis and decrease the immune recognition of EBV-associated disease [33,34,35]. Due to urgent need for specific and sensitive tumor markers for the early diagnosis and treatment response prediction of NPC, this study has a particular interest in developing a qPCR assay for early diagnosis and treatment response prediction of NPC patients based on detection of LMP1 30 bp deletion genetic biomarker.

The latent membrane protein 1 (LMP1) is one of the most important EBV latent proteins because it has been shown to induce phenotypic changes in both epithelial cells and B cells [36,37] and was reported to have an important role in NPC pathogenesis [38,39]. The significance of LMP1 in NPC tumorigenesis in vivo is confirmed by the observation that LMP1 was expressed in 78% of NPC samples [36,40]. The region of LMP1 thought to be essential for oncogenesis is C-terminus, a hot spot region for mutations such as 30 bp deletion that was reported as the most predominant deletion in C-terminus and was first observed in NPC patients from southern China [36,41].

The 30 bp deletion has been examined, and it has been shown that 30 bp deletion results in enhanced oncogenic behavior of infected cells and results in more aggressive EBV-associated tumor phenotypes [35,42]. An association between LMP1 30 bp deletion and the development of NPC was only observed in an Asian population, whereas no association in Europe and North Africa was observed [35,43,44]. The LMP1 30 bp deletion appears to be more predominant in NPC patients than in healthy individuals and in NPC-endemic regions rather than non-endemic regions [35,44]. The detection of this mutation will help in earlier diagnosis of NPC, particularly in early stages and suspected cases with higher sensitivity and specificity comparing with available molecular assays. In addition, this developed assay will help the clinicians in treatment response prediction, understand the extent of treatment effectiveness, and follow-up monitoring of NPC patients.

To the best of our knowledge, all available molecular assays to detect 30 bp deletion genetic biomarker were done using conventional PCR method, which are laborious, time-consuming and less sensitive method. Several advantages of qPCR over conventional PCR are well known, such as that it enables quantification, lower detection limit, higher reliability of assay and higher sensitivity and specificity [45,46]. Therefore, the aim of this study is to develop an analytic and diagnostic validated InnoPrimers-duplex real-time PCR (qPCR) for early detection of NPC blood circulating LMP1 30 bp deletion genetic biomarker with integration of amplification control for early detection of NPC and also for treatment response prediction of NPC patients.

## 2. Materials and Methods

### 2.1. Archive NPC Biopsy Tissue and FNA Samples

A total of 25 extracted genomic DNA from NPC patients were used for reference sequencing data and specificity analysis of the developed InnoPrimers-duplex qPCR. These samples were provided by the Department of Medical Microbiology and Parasitology, School of Medical Sciences, Universiti Sains Malaysia, Malaysia. The NPC archive biopsy tissue samples were nominated as AB samples in this study.

### 2.2. Reference Microorganisms’ Genomic DNA

A total of 48 bacterial, fungal and virus genomic DNA from both clinical isolates and ATCC (American Type Culture Collection) strains were used for specificity analysis. The reference microorganisms’ genomic DNA samples were obtained from Molecular Research Laboratory, Department of Medical Microbiology and Parasitology, School of Medical Sciences, Universiti Sains Malaysia, Malaysia. A 20 ng/µL of genomic DNA from each reference sample was used in this study unless specified otherwise.

### 2.3. Study Subjects

This current study involved 34 NPC patients who were enrolled in Hospital USM between October 2017 and July 2020, which included 6 suspected and newly diagnosed patients and 28 previously diagnosed NPC patients. The controls included 39 healthy individuals and 36 non-NPC cancer patients. All NPC suspected cases were confirmed by histopathological examination (HPE). The WHO NPC classification was used to determine the tumor pathological types [47]. The disease was restaged in accordance with the American Joint Committee of Cancer (AJCC) TNM staging method, seventh edition [48].

### 2.4. Whole Blood and Tissue Samples

A total of 109 EDTA (Ethylenediamine tetraacetic acid) whole blood (WB) samples from NPC cases (*n* = 34), non-NPC cancer patients (*n* = 36), healthy individuals (*n* = 39) and seven NPC tissue samples cases were included in this study. The NPC biopsy tissue samples from NPC cases were nominated as NB samples in this study.

### 2.5. Extraction of Genomic DNA

DNA from patients and healthy individuals’ WB and NB samples were extracted using the NucleoSpin^®^ Blood and NucleoSpin^®^ Tissue (MACHEREY-NAGEL GmbH & Co. KG, Germany, Germany) DNA extraction kit. The DNA extraction procedure was performed according to the manufacturer’s instruction with minor modifications to increase its yield by eluting the DNA in 50 µL pre-warmed TE buffer. Prior to the final centrifugation at 11,000× *g* for 1 min; the column was incubated at room temperature for 1 min. Total DNA was quantified using the Eppendorf BioPhotometer (Eppendorf Scientific, Inc., New York, NY, USA) and stored at −20 °C until use.

### 2.6. Design of PCR Primers and TaqMan Probe

The primers and probe of 30 bp deletion NPC genetic biomarker were designed using the IDT DNA PrimerQuest^®^ webtool (https://sg.idtdna.com/Primerquest/Home/Index; accessed on 12 June 2021) based on the nucleotide sequence of B95.8 prototype EBV genome (GenBank accessions no.: V01555.2, from 169,474 bp to 168,163 bp), which was retrieved from the National Center for Biotechnology Information (NCBI) database, and the sequencing results of 25 NPC AB and FNA samples to design the oligonucleotide sequences.

**Figure 1:**
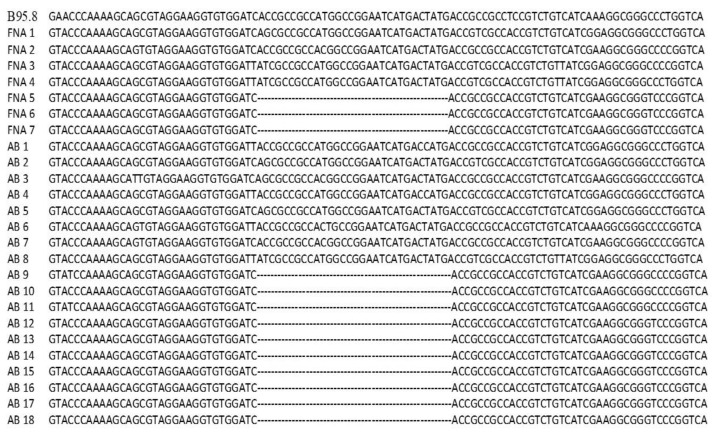
Alignment of reference strain (V01555.2) (first line) with 25 sequences of AB and FNA samples from NPC patients. Dot areas (…………………………) represent the location of 30 bp deletion.

**Figure 2:**
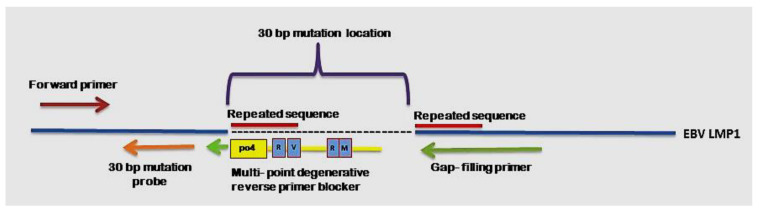
Schematic diagram of the InnoPrimers-duplex qPCR (innovative combination of the gap-filling mutant primer and multi-points degenerative reverse primer blocker) EBV LMP1 30 bp deletion assay.

The sequencing results were aligned with the B95.8 prototype EBV genome (reference sequence) to identify the selected 30 bp deletion region, as shown in Figure 1. In this study, the reverse primer to detect 30 bp deletion NPC genetic biomarker was assigned as gap-filling mutant primer due to its location before and after 30 bp deletion location, as shown in Figure 2. The sequences of primers and probes used are listed in Table 1.

### 2.7. Design of Non-Extendable Blocking Oligonucleotide

The non-extendable blocking oligonucleotide was designed based on standardized design rules by Morlan et al. with slight modifications [50]. In this study, the non-extendable blocking oligonucleotide was assigned as a multi-points degenerative blocker because it is a combination between blocking oligonucleotides and several degenerative bases. A multi-points degenerative blocker was designed to complement the wild-type sequence. Besides that, the addition of 3′ ends phosphorylated group aimed to inhibit 3′ exonucleases activity and prevent the extension of wild-type templates DNA polymerase, indirectly enhancing the PCR amplification of the mutant template. Four degenerative bases at 3′ end and in the middle of the blocker sequence were used to cover all possible combinations of WT nucleotide sequences.

The multi-points degenerative blocker was also designed to have a melting temperature higher than the qPCR cycling annealing temperature. The concentration of multi-points degenerative blocker was greater than the concentration of gap-filling mutant primer. The location of multi-points degenerative blocker was shown in Figure 2. The sequence of multi-points degenerative blocker used is listed in Table 1.

### 2.8. Designing of Synthetic DNA

The synthetic double-strand DNA (dsDNA) or called gBLOCKs^®^ Gene Fragments for both LMP1 MT (mutant type, have 30 bp deletion; named gBLOCK MT) and LMP1 WT (wild-type, have 30 bp sequence region; named gBLOCK WT) were used as a positive control for both MT and WT variants, respectively. The design of the gBLOCKs was based on B95.8 prototype EBV genome (GenBank accessions no.: V01555.2, from 169,474 bp to 168,163 bp) and sequencing results of retrospective NPC AB and FNA samples.

The synthetic dsDNA for Internal Application Control (IAC) oligonucleotides was designed by a previous study based on the fusion of 83–110 bp region of *Entamoeba histolytica HLY5mcl* gene (Accessions no. Z29969.1) and *Mycobacterium tuberculosis rpoB* gene (Accessions no. NC_000962.3:759807-763325) [49]. The IAC was incorporated to rule out the false negative result.

### 2.9. Optimization of qPCR Master Mix

Optimization of InnoPrimers-duplex qPCR assay was conducted by testing the different concentration of target’s probe (100–400 nM), primers (100–500 nM), MT gBLOCK (20 pg–2 fg per reaction), WT gBLOCK (200 fg–20 ag per reaction) and IAC synthetic DNA (20 fg–20 ag per reaction). Moreover, the ratios of gap-filling mutant primer:multi-points degenerative blocker ranging from 1:4 to 1:120 were tested in the optimization process of this developed assay. The optimal concentrations of synthetic DNA and oligonucleotides were used in this developed qPCR assay.

### 2.10. Optimization of Thermal Cycling Condition

The PCR reactions was conducted according to Luminaris Probe qPCR master mix (standard annealing temperature is 60 °C) with slight modification on annealing temperature (different temperatures were tested ranged from 55 °C to 61 °C). The optimal annealing temperature were used in this developed qPCR assay.

### 2.11. Analytical Sensitivity Analysis

The analytical sensitivity was conducted using a 10-fold serial of MT gBLOCK DNA templates (synthetic DNA) in the range of 1 ng/µL to 1 atto/µL as a template, as shown in Table 2. InnoPrimers-duplex qPCR was performed in triplicates, using 2 μL of each dilution. The limit of detection (LOD) was determined as the lowest amount of MT gBLOCK DNA that could be detected by at least one of the triplicates. The number of DNA copies was calculated based on an online tool developed by Andrew Staroscik from the URI Genomics and Sequencing Centre (available at http://cels.uri.edu/gsc/cndna.html; accessed on 25 June 2021). The formula is as follows:

**DNA copies number** = ((DNA concentration (ng) × 6.022 × 10^23^))/((DNA template length (bp) × 1 × 10^9^ (ng/g) × 650(g/mole of bp)). (1)

### 2.12. Analytical Specificity Analysis

The analytical specificity was conducted using 2 μL DNA from 21 fungal strains, 17 bacterial strains, 5 CMV (viral load ranged between 702 IU/mL (intermediate) and 45,474 IU/mL (high)) and 5 HPV (concentrations range 46 ng/µL–10 pg/µL), which were used as a template. Details of the reference microorganisms’ genomic DNA are listed in Table 3. In addition, 12 of AB and FNA samples from NPC patients were used to evaluate the analytical specificity of the InnoPrimers-duplex qPCR as listed in Table 4.

### 2.13. Diagnostic Evaluation

The diagnostic evaluation was done based on the detection of 30 bp deletion NPC genetic biomarker in WB samples of newly diagnosed and suspected NPC cases (*n* = 6), non-NPC cancer patients (*n* = 36) and healthy individuals (*n* = 39). The diagnostic performance of the InnoPrimers-duplex qPCR assay was evaluated based on clinician diagnosis. In addition, the conventional PCR method was used in this study for diagnostic evaluation [36]. The diagnostic evaluation of the developed InnoPrimers-duplex qPCR assay included diagnostic specificity, diagnostic sensitivity, negative predictive value (NPV) and positive predictive value (PPV). The following formulas were used for diagnostic evaluation:

(2) Diagnostic sensitivity=true positive/(true positive + false negative) 

(3) Diagnostic specificity=true negative/(true negative + false positive) 

(4) PPV = true positive/(true positive + false positive)

(5) NPV = true negative/(true negative + false negative)

### 2.14. Treatment Response Prediction of NPC Patients

The NPC patients who were diagnosed with advance stages had received concurrent chemoradiation therapy (CCRT), and patients with early stages had received radiotherapy alone, as reported by other literatures [51,52]. In this study, the treatment response prediction of this assay was investigated using WB samples from 34 NPC patients. The clinician treatment response prediction was based on the patient-related factors, TNM classification, histological type, staging, follow-up imaging results (MRI, CT and PET/CT scan), treatment-related factors and clinical examination of the patients. In addition, RECIST guideline was used in clinician treatment response prediction, which is a commonly used guideline in various regularity authorities for the assessment of tumor outcome [53,54]. The pathological biopsy was performed for the suspected recurrent NPC patients to verify the progression of the locoregional or distant metastasis. Based on RECIST criteria, NPC patients were classified as complete response (CR), partial response (PR) and progressive disease (PD)/recurrence [53,54]. The evaluation of 30 bp deletion genetic biomarker as a predictor for treatment response was done by comparing treatment response prediction of the 30 bp deletion genetic biomarker with clinician treatment response prediction.

### 2.15. Data Analysis and Interpretation of Results

The preliminary specificity of the primers and probes was assessed using the Primer-BLAST webtool (http://blast.ncbi.nlm.nih.gov/Blast.cgi; accessed on 12 July 2021). The mean Cq and standard deviation were calculated from each of the Cq values obtained in the replicates. Baseline was set at 25 RFU and was adjusted and analyzed using the CFX Manager™ Software. PCR amplification efficiency, E, was calculated using this formula:

E = 10^−1/slope^ − 1 (6)

The data analyzed using SPSS (statistical package of social science) version 24.0. Person Chi-square (χ2) and Fisher exact tests were used to analyze the association between 30 bp deletion genetic biomarker and clinical outcomes (treatment response prediction). *p*-values less than 0.05 were considered to indicate a significant association.

## 3. Results

### 3.1. NPC Patients’ Characteristics

The majority of NPC patients were Malay, followed by Chinese race (88.2% and 11.9%, respectively). Most of the NPC patients were diagnosed with WHO type III (*n* = 19, 55.9%), followed by WHO type II (*n* = 12, 35.3%), WHO type I (*n* = 2, 5.9%) and papillary variant (*n* = 1, 2.9%). The locally advanced NPC stages were found in the vast majority of NPC patients (stage IV and stage III), where 55.9% (*n* = 19) of NPC patients had stage IV and 41.2% (*n* = 14) had stage III. However, one patient reported as stage II (*n* = 1). All NPC patients received CCRT, except three patients (suspected cases) who were not receiving any treatment at time of sample collection. The suspected NPC patients were confirmed to have NPC disease by HPE and regular diagnostic procedures such as a serological test, imagining techniques, full blood count and biochemistry profile.

### 3.2. Design and Evaluation of Primers, Non-Extendable Blocking Oligonucleotide and Probes

**Figure 3:**
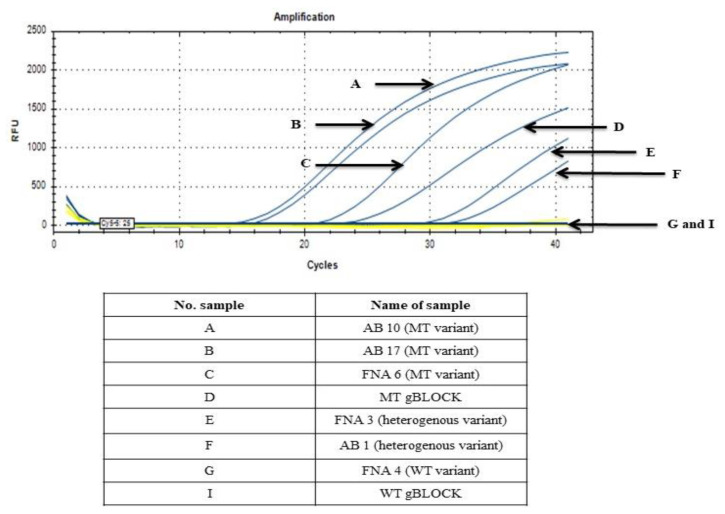
Preliminary functionality of the InnoPrimers-duplex qPCR for detecting of 30 bp deletion NPC genetic biomarker among MT gBLOCK, WT gBLOCK, NPC AB and FNA samples. Abbreviations: AB, archive biopsy sample; FNA, fine needle aspiration.

A set of primers and a probe were successfully designed based on the nucleotide sequence of B95.8 prototype EBV genome (GenBank accessions no.: V01555.2) and the sequencing results of 25 NPC AB and FNA samples, as shown in Figure 1 and Figure 2. The preliminary functionality of the designed primer and probe was investigated using the MT gBLOCK and WT gBLOCK, which were used as PCR positive control for LMP1 MT and LMP1 WT templates, respectively, and AB and FNA samples from NPC patients by InnoPrimers-duplex qPCR platform to detect 30 bp deletion NPC genetic biomarker. Preliminary functionality of the InnoPrimers-duplex qPCR for detecting of 30 bp deletion NPC genetic biomarker is shown in Figure 3.

### 3.3. Optimization of qPCR Parameters

The optimal concentration of target’s probe, primers, MT gBLOCK, WT gBLOCK and IAC synthetic DNA, 400 nM, 500 nM, 20 fg, 20 fg and 60 ag, respectively, were selected in this study. The annealing temperature of 60 °C was chosen as an optimal temperature; this temperature is recommended by Luminaris Probe qPCR Master Mix protocol. A ratio of 40:1 was selected as an optimal ratio of multi-points degenerative blocker:gap-filling mutant primer in this developed assay.

### 3.4. The InnoPrimers-Duplex qPCR Parameters and Thermal Cycling Condition

The PCR reaction contained 1 × Luminaris Probe qPCR Master Mixes (Thermo Scientific, Waltham, MA, USA), 500 nM of target’s forward primer, 500 nM gap-filling mutant primer, 20 µM of multi-points degenerative blocker, 400 nM target’s probe, 7 μL DNA template and PCR grade water, adjusted to a total volume of 20 μL. To exclude the effect of the inhibitor, IAC was used in this study, where 300 nM of IAC’s forward primer, 300 nM of IAC’s reverse primer and 200 nM IA’s probe were incorporated in the same PCR reaction. Sequences of oligonucleotides used are listed in Table 1. The reaction was subjected to UDG pre-treatment at 50 °C for 2 min; an initial denaturation step at 95 °C for 10 min; 40 cycles of 95 °C for 15 s and 60 °C for 30 s.

Baseline threshold for the post-amplification analysis was set at 25 for both IAC and 30 bp deletion NPC genetic biomarker. Positivity was determined at any quantification cycle, Cq value < 37 (for IAC), <37 (for 30 bp deletion NPC genetic biomarker in WB samples) and <27 (for 30 bp deletion NPC genetic biomarker in NB samples). All the assays were carried out in three replicates, unless specified otherwise, using CFX 96 Touch™ Real-Time PCR Detection System.

### 3.5. Analytical Sensitivity and Specificity of the Developed InnoPrimers-Duplex qPCR Assay

**Figure 4:**
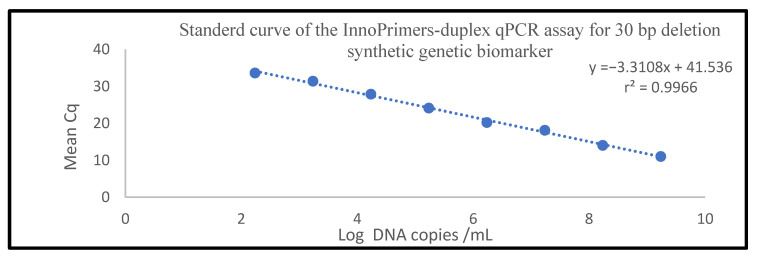
Standard curve of the InnoPrimers-duplex qPCR showing amplification of 10-fold dilutions of 30 bp deletion synthetic DNA NPC genetic biomarker (MT gBLOCK).

The analytical evaluation showed a linear relationship, with a regression coefficient (r^2^) value of 0.9966 (Figure 4). The InnoPrimers-duplex qPCR was able to amplify 20 ag DNA per reaction, which was 173 copies/reaction, equivalent to 8650 copies/mL, in all replicates (Table 2). At 2 ag per reaction (equivalent to ~17.3 copies/reaction), one of the replicates was found to be positive (Table 2). The PCR efficiency of the InnoPrimers-duplex qPCR was 100% for 30 bp deletion NPC genetic biomarker in this study, which represented a twofold increase in the amplicon’s level after each cycle, and this efficiency value was within the acceptable range, as the acceptable range of efficiency is 90–120% [55,56].

**Figure 5:**
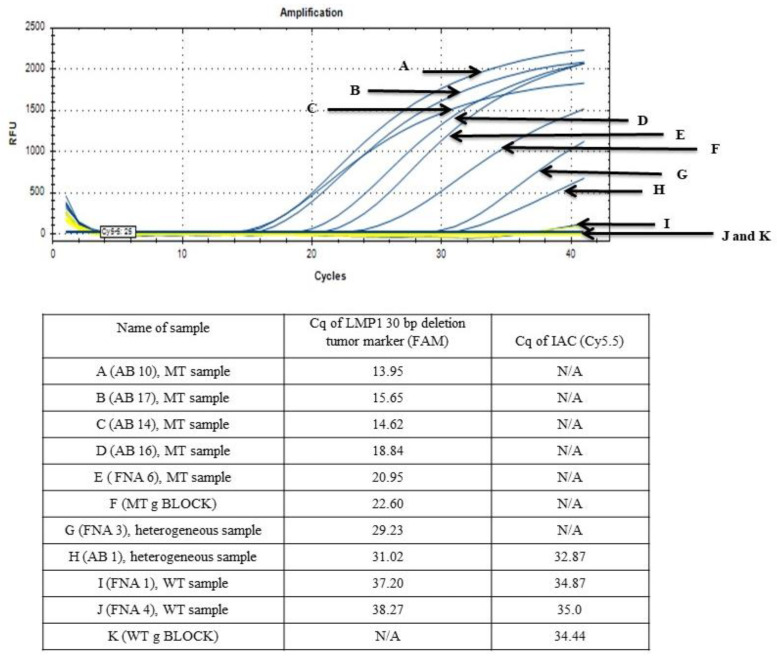
The functionality of the developed InnoPrimers-duplex qPCR. Abbreviations: AB, archive biopsy sample; FNA, fine needle aspiration.

The assay amplified the 30 bp deletion NPC genetic biomarker in all MT variant FNA and AB samples from NPC patients, and this developed assay was able to differentiate between WT variant from MT variant among NPC AB and FNA samples, as shown in Figure 5. No undesired amplification was observed in the qPCR reactions with other reference microorganisms’ genomic DNA (all samples had Cq ≥ 37), as listed in Table 3. The conventional PCR, sequencing and InnoPrimer-duplex qPCR results of 30 bp deletion NPC genetic biomarker among AB and FNA samples were found to be comparable, where all MT variants had Cq < 27, while WT and heterogeneous variants had Cq ≥ 27, as shown in Figure 5 and Table 4. In heterogeneous samples, if the presence of the MT variant is more predominant over the WT type variant in the same samples, then the Cq values will be lower than 27, which is also depends on the clinical situation of the NPC patient.

The number of amplification cycles necessary for the target gene to surpass a threshold level is represented by real-time RT-PCR cycle threshold (Cq) values. The Cq values are, therefore, inversely related to viral load and can provide an indirect approach of quantifying viral RNA copy number in the sample [57].

### 3.6. Diagnostic Sensitivity and Specificity of the Developed InnoPrimers-Duplex qPCR Assay

The diagnostic evaluation of this developed qPCR assay was done using six NPC (newly diagnosed and suspected cases) patients, non-NPC cancer patients and healthy individuals. The diagnostic sensitivity and specificity of this assay were 83.3% and 100%, respectively. High PPV and NPV were observed with 100% and 98.7%, respectively.

### 3.7. Detection of 30 bp Deletion NPC Genetic Biomarker in Healthy, Non-NPC Cancer and NPC Samples

The presence of LMP1 30 pb deletion was investigated in all patients’ and healthy individuals’ blood and NB samples by a conventional PCR method as a standard method [36]. The conventional PCR result and the InnoPrimers-duplex qPCR results was compatible among NPC samples, where all WB samples with WT variant (*n* = 13/13) had Cq ≥ 37, and WB sample with MT variant (*n* = 1/1) had Cq < 37. In addition, all tissue samples with WT variant (*n* = 2/2) had Cq ≥ 27, and tissue samples with MT variant (*n* = 2/2) had Cq < 27. In contrast, heterogeneous samples had different Cq values depending on the spectrum and varying proportions of these two variants in the NPC WB and NB samples. Among NPC WB samples with heterogenous variant, 50% (*n* = 10/20) of samples had Cq ≥ 37, and 50% (*n* = 10/20) of samples had Cq < 37. Among NB samples with heterogenous variant, 67% (*n* = 2/3) of samples had Cq ≥ 27, and 33% (*n* = 1/3) of samples had Cq < 27.

On the other hand, all non-NPC cancer WB samples (*n* = 36) and healthy WB samples (*n* = 0/39) were not detected with 30 bp deletion NPC genetic biomarker (Cq ≥ 37), while IAC was amplified in all the tested clinical samples.

### 3.8. Treatment Response Prediction of the Developed InnoPrimers-Duplex qPCR

Different cut-off Cq values were set for 30 bp deletion genetic biomarker to predict the NPC patients’ treatment response in both WB and tissue samples. Among NPC WB samples, Cq ≥ 37 indicated CR, Cq value ranging from 36.99 to 32.00 indicated PR and Cq ≤ 31.99 indicated PD/recurrence. The evaluation of 30 bp deletion genetic biomarker detection as a predictor for treatment response is shown in Table 5.

Based on treatment response prediction of clinician and 30 bp deletion genetic biomarker, the majority of NPC patients showed CR (*n* = 15/34), while five patients showed PR and two patients showed PD/recurrence. Among 19 patients who were diagnosed as WHO type III, 12 patients had CR. However, among 12 patients were diagnosed as WHO type II, 5 patients had CR and among 2 patients were diagnosed as WHO type I, 1 patient had CR. These results indicated that the WHO type III had a better prognosis and response to treatment compared with WHO type II and type I. The 30 bp deletion genetic biomarker was found to be a good prognostic biomarker associated with overall clinical outcome of NPC WHO type III patients more than WHO type II and I (Table 5).

A significant association between clinician treatment response prediction and Cq values of 30 bp deletion genetic biomarker (*p* = 0.033) was detected (Table 6). More than half of NPC patients who showed a response to treatment (including 44.2% who had CR and 14.7% who had PR) had Cq values of more than 37. Therefore, a 30 bp deletion genetic biomarker was shown as a good prognostic biomarker or predictor for treatment response and for NPC patients’ overall clinical outcome.

## 4. Discussion

In this study, most NPC patients were Malay (88.2%), as reported by other studies performed in Malaysia and Singapore [58,59]. NKC constituted about 91.2% (55.9% WHO type III and 35.3% WHO type II), and 5.9% of NPC patients had squamous cell carcinoma (SCC) (WHO type I) in this study, which was comparable to the previous studies, where they reported higher occurrence rates of NKUC followed by NKDC and lower rates of SCC [58,60,61,62]. One patient was diagnosed as papillary variant, nasopharyngeal adenocarcinomas (NACs), which is a rare tumor accounting for less than 0.5% of nasopharyngeal malignancies and can occur in up to 10% of NPC cases in the endemic areas [63,64]. The vast majority of NPC patients in this study had locally advanced NPC (stage IV and stage III). Approximately half of the patients had stage IV, and 41.2% had stage III. These findings were quietly comparable with other Malaysia studies [58,60,62,65].

The early diagnosis of NPC is critical and essential in the refined treatment and improved prognosis of NPC patients [10,13]. Due to the narrow spectrum and non-specific early symptoms during the early stages of NPC, the clinicians and the patients can easily miss them [2,10]. Despite recent progress in diagnostic techniques (such as serology, imaging and fiberoptic nasopharyngoscopy), only around 10% of all new NPC cases can be detected in the early stages) [10].

Finding of biomarkers for early detection, prediction of metastasis and recurrence and NPC therapeutic monitoring is of great importance to guide NPC treatment and improve patient prognosis [66]. LMP1 is called an oncogene “all-in-one” was designed during viral evolution because LMP1 functions as a classic oncogene and is necessary for immortalization and the transformation of B cells and has the ability to encourage proliferation and to antagonize apoptosis and senescence [37,67,68]. A previous researches reported a significant correlation between the LMP1 expression and treatment response where LMP1 encouraged metastasis and decreased the survival [32,69]. Moreover, there was a significant difference in 24-month survival between LMP1 (+) NPCs relative to LMP1 (−) NPCs [32]. The LMP1 was reported to play a crucial role in the outcome of treatment, which is also consistent with the hypothesis that stated that LMP1 is an anti-apoptotic factor that affects tumor resistance to anti-tumor drugs [69,70,71,72].

It has been shown that 30 bp deletion is a prominent polymorphism in LMP1 that results in a progression from non-oncogenic to oncogenic and in more aggressive EBV-associated tumor phenotypes [73,74]. In addition, recent studies have shown that some of the LMP1 gene sequence variations, such as 30 bp deletion, are linked to increased tumorigenicity and decreased immune recognition [39,40]. Several studies have investigated the occurrence of EBV LMP1 30 bp deletion in different types of EBV-associated cancers using sequencing and conventional PCR methods, mainly in invasive biopsy samples and plasma samples [35,43,44,75]. A simple, time-saving, less invasive, cost-effective, efficient, early diagnostic and prognostic method is required in the clinical setting. To the best of our knowledge, no qPCR assay is available to detect EBV’s LMP1 30 bp deletion in WB and NB samples from EBV-associated NPC patients. Therefore, this study aimed to develop a Taqman probe-based qPCR to detect the 30 bp deletion genetic biomarker for early diagnosis and treatment response prediction of NPC patients.

This study aimed to develop a prototype innovative qPCR assay based on utilizing the unique features and innovative combination of the “gap-filling mutant primer” with “multi-points degenerative blocker” technology to detect EBV LMP1 30 bp deletion (genetic biomarker) from a less invasive WB sample and from NB sample from NPC patients. The purpose of using the multi-points degenerative blocker in this study was to reduce wild-type templates amplification to undetectable levels, even when the presence of WT templates exceeds the presence of the MT template by a 1000-fold [52,76,77]. In addition, our purpose was to increase the specificity, sensitivity and selectivity of the InnoPrimers-duplex qPCR assay.

In terms of analytical sensitivity, this developed qPCR assay was capable of detecting the 30 bp deletion NPC genetic biomarkers as low as 173 copies/reaction; taking into account this study’s nucleic acid extraction and PCR set-up, 173 copies/reaction is equivalent to 8650 copies/mL. To the best of our knowledge, there is no available qPCR study to detect the presence of 30 bp deletion NPC genetic biomarkers and to determine the LOD of 30 bp deletion NPC genetic biomarkers, particularly in WB samples from NPC patients. Hence, to the best of our knowledge, this assay is the first semi-quantitative qPCR assay to detect LMP1 30 bp deletion in WB and NB samples from NPC patients. A previous study reported the LOD of LMP1 as 50 copies/PCR reaction [78] and LOD of LMP genes as 200 copies/mL (EBV Real-TM Quant kit) [26]. The viral load of 5000 copies/mL of plasma EBV DNA was reported as the median amount for early stage NPC patients [79]. Meanwhile, the median plasma EBV DNA concentrations were found to be eightfold higher in advanced stage NPC patients relative to early stage NPC patients [80]. Among all anatomical NPC stages, the median concentration of EBV DNA ranged between 9 and 82,500 copies/mL [81]. The InnoPrimers-duplex qPCR assay is expected to be able to detect 30 bp deletion NPC genetic biomarker within this range.

The assays linearity was reported based on r^2^ values (near to 1). In this developed assay, r^2^ values were 0.9966, which was close to 1. The PCR efficiency of the InnoPrimers-duplex qPCR was 100% for detection of 30 bp detection NPC genetic biomarker, which represented a twofold increase in the amplicon’s level after each cycle, and this efficiency value was within the acceptable range, as the acceptable range of efficiency is 90–120% [55,56,82]. This developed assay is very specific for NPC disease with 100% of specificity, where the 30 bp deletion NPC genetic biomarker was not detected among all non-NPC patients and healthy individuals. In this study, the sensitivity of detecting 30 bp deletion NPC genetic biomarker among suspected and newly diagnosed NPC patients was 83.3% across all anatomical stages, which was comparable with a previous study in China [83]. Review studies reported sensitivities ranged from 27% to 96%, and specificities ranged from 38% to 100% of detection of EBV DNA (other than 30 bp deletion NPC genetic biomarker) in plasma and serum of NPC [20,22,84]. However, the proportion of NPC patients with elevated blood or plasma levels of EBV DNA varies between studies, and procedures are not well standardized [85].

In this study, among NPC patients with heterogenous variant, half of WB samples had Cq < 37, and 33% of tissue samples had Cq < 27. In heterogeneous NPC WB and NB samples, if the presence of the MT variant is more predominant over the WT type variant in the same samples, then the Cq values will be lower than 37 (WB sample) and lower than 27 (NB sample), which also depends on the clinical situation of the NPC patient. It was speculated that both variants are derived from the single EBV strain during clonal proliferation of EBV-infected cells over a period of time or could be resulted from several EBV infections by more than one strain [86,87]. Heterogeneous-type cases with high Cq of 30 bp deletion NPC genetic biomarker could be related to the aggregation of LMP1 30 bp deletion after repeated cycles of clonal proliferation over a long period, where MT variants are exceeding WT variants owing to selection pressure. This hypothesis may explain the spectrum and varying proportion of these two variants; hence, different Cq values occurred in different samples. Moreover, the presence of 30 bp deletion NPC genetic biomarker in WB samples was more likely derived from the tissue by comparing the paired samples (WB and tissue) from the same patient, as reported in a previous study by Chan et al., 2003 [88]. In this study, the WB sampling procedure is considered a less invasive, easy, only producing slight discomfort, well-tolerated method and was useful in early NPC diagnosis, treatment response monitoring and evaluation of NPC progression, thereby minimizing the need for invasive biopsies.

This study finding showed that this developed assay was able to predict treatment response for WHO type III NPC patients more than type WHO II and type I NPC patients. The possible reason for that is the strong association between WHO type III NPC patients and EBV compared with the other two types [89]. Moreover, a previous study reported significantly higher EBV DNA contents in WHO type III NPCs and lower EBV DNA contents in WHO type I and WHO type II NPCs. These data suggested that EBV DNA replication was favorable in undifferentiated epithelial cells [90]. This relationship with EBV is essential not only for epidemiological reasons or diagnosis but also for patient monitoring, prognosis and therapeutic strategies [91]. Although WHO type III NPC is highly invasive and metastatic type [92], WHO type III was found to be responsive to chemotherapy and radiotherapy where a better prognosis was found in patients with WHO type III than with WHO type I or II [89,93]. On the other hand, differentiated NPC that is not associated with EBV infection exhibits comparable characteristics to the head and neck cancers. Compared with EBV-associated NPC, EBV-non-associated NPC possesses fewer chemo-radiosensitive properties and is locoregionally aggressive and highly metastatic [92]. These findings justify this study finding, where 58.9% of NPC patients showed response to treatment (including 44.2% that had CR and 14.7% that had PR).

In this study, three NPC patients were detected with Cq values ≥37, but based on the clinician treatment response prediction, all three patients had PD/recurrence status. There are several possible reasons for this discordant prediction. First, it may be that using a relatively small WB sample volume (200 µL) in the DNA extraction method may affect this genetic biomarker’s detection rate, related to using a small amount of extracted EBV DNA in qPCR assay [22]. The second reason is the instability of circulating EBV DNA that had been detected previously in several studies that resulted in low sensitivity in EBV-positive cases and highlighted the restrictive parameters of conservation of specimens that create practical and logistic challenges [94,95]. The third potential explanation is that the concentration of EBV DNA after treatment will decrease with a mean half-life of 6.3 days (ranging from 1 to >200 days) [96] or 3.8 days (interquartile range, 2.4–4.4 days) [97]. The fourth possible reason is that fluorodeoxyglucose–positron emission tomography (FDG–PET)/computed tomography (CT) scan can lead to a false-positive result because of increased FDG absorption and can quickly confuse an inflammatory reaction. Moreover, the CT and MRI specificity in the diagnosis of recurrent nasopharyngeal disease was low, with 59% and 76%, respectively [93,98].

On the other hand, one patient had detected with Cq value of 28.96, but based on clinician treatment response prediction, this patient had CR. The possible reason for this discordant prediction was the insufficiency of current clinical information and the imaging results (lost to follow-up). The second reason was that the patient was asymptomatic in the last clinical follow-up and had a T2 stage (minimal tumor volume) that may not be detected during physical examination and nasopharyngoscopy. The third possible reason is that the CT and magnetic resonance imaging (MRI) sensitivity in the diagnosis of the residual or recurrent nasopharyngeal disease is limited, as reported previously, with 76% and 78%, respectively [93,98]. The fourth possible reason is if this patient had a recurrence or metastasis to the mediastinal lymph nodes or had benign lesions, such as cystic hepatic lesions. These clinical situations are associated with a prolonged duration of elevated EBV DNA level, but no apparent recurrence has been established [12,99,100,101]. This patient requires long-term follow-up, and these findings should be considered during the follow-up of NPC patients with extended elevation of EBV DNA.

A significant association between clinician treatment response prediction and categorical Cq values of 30 bp deletion was reported in the current study, where 87% (20/23) of the patients who were detected with Cq ≥ 37 (*n* = 23) showed CR and PR after CCRT, 65.2% and 21.8%, respectively, and among three patients who had Cq ≤ 31.99, two (66.7%) patients showed PD/recurrence. These findings were comparable with other studies [102,103,104,105,106,107,108,109]. They reported that the patients with NPC that remained in remission following radiotherapy had regularly undetectable or extremely low plasma EBV DNA levels. In contrast, patients with developed recurrence had dramatically increased plasma EBV DNA levels; hence, EBV DNA load plays an important role in diagnosing and monitoring recurrence in NPC [102,103,104,105,106,107,108,109]. These findings indicate that the 30 bp deletion genetic biomarker analysis may be beneficial to classify patients to more likely benefit from more intensive therapy and to save other patients from unnecessary treatments and excessive economic strain.

The prognostic significance of pre-treatment and post-treatment plasma EBV DNA levels has been validated in different studies [110,111,112,113,114]. The timing of sample collection is not consistently specified in many published studies. Some evaluate EBV DNA before treatment initiation [111] or during therapy [115], and others, directly after completion of treatment [19,113,116]. This lack of standardization in the specimen’s collection timing can contribute to inconsistency in the reported post-treatment EBV DNA levels, although some patients with consistently undetectable plasma EBV DNA still develop tumor recurrence during post-treatment follow-up. In contrast, some patients with elevated EBV DNA levels stay disease-free, even after long-term follow-up [99]. Thus, the recurrence rates for patients with undetectable or detectable plasma EBV DNA during the post-treatment follow-up phase remain unknown [117]. In addition, it was also known that not all NPC patients were detected with EBV DNA. The reported sensitivities ranged between 53% and 96%, according to analysis of 15 studies involved the quantitation of EBV DNA [22]. Until now, the best specimens for measuring viral loads and the threshold value for the medical intervention and the measurement units are also still questionable and not standardized [118]. Consequently, the recommendations for the diagnosis of EBV-associated diseases or initiation of treatment are unclear.

Overall, the development of this qPCR assay can successfully detect the 30 bp deletion NPC genetic biomarker and differentiate between WT and MT variants among NPC patients and is also able to distinguish NPC from among both non-NPC cancer and healthy individuals. In addition, this developed qPCR assay may help the clinician in early diagnosis, determining the intervention appropriateness, treatment response prediction, extent of treatment effectiveness, post-treatment follow-up monitoring and prognosis of NPC. Further clinical evaluation should be carried out on a larger cohort using this developed molecular assay. Prior to clinical evaluation, further analytical validation, such as intra- and inter-assay variation, higher number of replicates and optimization of assays are necessary. Although our study tested a limited number of samples, the high sensitivity and specificity of the InnoPrimers-duplex qPCR is favorable for a future study with a larger number of clinical specimens.

## 5. Conclusions

This developed InnoPrimers-duplex qPCR assay is a sensitive, specific, time-saving (results ready within 2 h) and simple method that utilized less invasive and minimally traumatizing WB sampling methods, which will be the best alternative to an invasive biopsy sampling method in the future. This developed qPCR assay shows high specificity in detection of the LMP1 30 bp deletion genetic biomarker among NPC patients, where this assay is capable of differentiating between MT and WT variants in NPC samples and is able to distinguish NPC from among both non-NPC cancer and healthy individuals. The 30 bp deletion genetic biomarker was found to be a good prognostic biomarker associated with overall clinical outcome of NPC WHO type III patients. Even though the number of tested clinical samples is limited, it provides crucial preliminary data for a subsequent larger scale study in Malaysia.

## Figures and Tables

**Table 1:** List of primers, multi-points degenerative blocker and probes used in this study.

Target Gene		Sequences (5′→3′ End)	Source
LMP1 30 bp deletion	Forward primer	GTCATAGTAGCTTAGCTGAAC	This study
	Gap-filling primer (reverse primer)	ACGGTGGCGGCGGTG	This study
	Multi-points degenerative blocker (reverse sequence)	CGGCMRTGGCGGCGVTR-PO4 *	This study
	Reverse probe	FAM-ATCCACACCTTCCTACGCTGCTTTTGG-BHQ_1	This study
IAC	Forward primer	AAGGAGTTCTTCGGCACCA	[49]
	Reverse primer	GGCGCTTGTGGGTCAAC	[49]
	Forward probe	Cy5.5-TTCCTGCTATTCTCATTCGCATCCATGT-IBRQ	[49]

* Oligonucleotide label M refers to A or C base, R refers to A or G base and V refers to A or C or G base.

**Table 2:** Mean threshold value (Cq), coefficient of variation (CV) and standard deviation (SD) for duplex developed InnoPrimers-duplex qPCR to determine the LOD of 30 bp deletion NPC genetic biomarker.

Concentration	Copies Number	Copies/mL	Number of Replicates	Number of Positive	Developed InnoPrimers-Duplex qPCR		
					Mean Cq	SD	CV%
2 ng	17,300,000,000	865 × 10^9^	3	1	2.73	4.73	173.21
200 pg	1,730,000,000	865 × 10^8^	3	3	11.02	0.37	3.31
20 pg	173,000,000	865 × 10^7^	3	3	14.01	0.61	4.37
2 pg	17,300,000	865 × 10^6^	3	3	18.11	0.25	1.36
200 fg	1,730,000	865 × 10^5^	3	3	20.21	2.30	11.40
20 fg	173,000	865 × 10^4^	3	3	24.11	0.97	4.03
2 fg	17,300	865 × 10^3^	3	3	28.86	0.41	1.46
200 ag	1730	865 × 10^2^	3	3	31.39	1.21	3.85
20 ag	173	8650	3	3	33.6	0.12	0.34
2 ag	17.3	865	3	1	39.36	1.11	2.81
200 zg	1.73	86.5	3	0	-	-	-

**Table 3:** List of reference microorganisms’ genomic DNA used in this developed assay.

Microorganism (*n* = 48)	Source	Origin	No. Tested	Result of LMP1 30 bp Deletion NPC Genetic Biomarker by the InnoPrimers-Duplex qPCR Assay
*Aspergillus flavus*	Clinical isolate	HUSM	1	Negative
*Aspergillus fumigatus*	Clinical isolate	HUSM	1	Negative
*Aspergillus nidulans*	Clinical isolate	HUSM	1	Negative
*Aspergillus niger*	Clinical isolate	HUSM	1	Negative
*Aspergillus terreus*	Clinical isolate	HUSM	1	Negative
*Bacillus subtilits*	Clinical isolate	HUSM	1	Negative
*Candida* *albican*	Clinical isolate	HUSM	1	Negative
*Candida famata*	Clinical isolate	HUSM	1	Negative
*Candida glabrata*	Clinical isolate	HUSM	1	Negative
*Candida* *krusei*	Clinical isolate	HUSM	1	Negative
*Candida lusitaniae*	Clinical isolate	HUSM	1	Negative
*Candida parapsilosis*	Clinical isolate	HUSM	1	Negative
*Candida rugosa*	Clinical isolate	HUSM	1	Negative
*Candida tropicalis*	Clinical isolate	HUSM	1	Negative
*Cladosporium* spp.	Clinical isolate	HUSM	1	Negative
*Corynebacterium diphtheria*	Clinical isolate	HUSM	1	Negative
*Cryptococcus neoformans*	Clinical isolate	HUSM	1	Negative
*Cytomegalovirus* (CMV)	Clinical isolate	HUSM	5	Negative
*Enterobacter cloacae*	Clinical isolate	HUSM	1	Negative
*Enterobacter sp.*	Clinical isolate	HUSM	1	Negative
*Escherichia coli*	ATCC (25922)	HUSM	1	Negative
*Fusarium oxysporum*	Clinical isolate	HUSM	1	Negative
*Fusarium proliferatum*	Clinical isolate	HUSM	1	Negative
*Haemophilus influenza*	ATCC (49247)	HUSM	1	Negative
Human papillomavirus (HPV)	Clinical isolate	HUSM	5	Negative
*Klebsiella pneumoniae*	Clinical isolate	HUSM	1	Negative
*Klebsiella oxytoca*	Clinical isolate	HUSM	1	Negative
*Neisseria meningitides*	ATCC (13090)	HUSM	1	Negative
*Penicillium marneffei*	Clinical isolate	HUSM	1	Negative
*Pseudomonas aeruginosa*	ATCC (27853)	HUSM	1	Negative
*Rhizopus* spp.	Clinical isolate	HUSM	1	Negative
*Scedosporium aurantiacum*	Clinical isolate	HUSM	1	Negative
*Staphylococcus aureus*	ATCC (25923)	HUSM	1	Negative
*Staphylococcus epidermidis*	ATCC (12228)	HUSM	1	Negative
*Stenotrophomonas maltophilia*	Clinical isolate	HUSM	1	Negative
*Streptococcus Group A*	Clinical isolate	HUSM	1	Negative
*Streptococcus mitis*	Clinical isolate	HUSM	1	Negative
*Streptococcus pneumonia*	Clinical isolate	HUSM	1	Negative
*Trichophyton rubrum*	Clinical isolate	HUSM	1	Negative
*Trichosporon asahii*	Clinical isolate	HUSM	1	Negative

**Table 4:** The analytical specificity of the InnoPrimers-duplex qPCR assay among AB and FNA samples from NPC patients.

Name of Sample	Source	Origin	Sequencing Results	Conventional PCR Results	Result of LMP1 30 bp Deletion NPC Genetic Biomarker in Developed Assay
AB 1	NPC	HUSM	WT	Heterogenous type	Negative
AB 9	NPC	HUSM	MT	MT	Positive
AB 10	NPC	HUSM	MT	MT	Positive
AB 12	NPC	HUSM	MT	MT	Positive
AB 14	NPC	HUSM	MT	MT	Positive
AB 16	NPC	HUSM	MT	MT	Positive
AB 17	NPC	HUSM	MT	MT	Positive
FNA 1	NPC	HUSM	WT	WT	Negative
FNA 3	NPC	HUSM	WT	Heterogenous type	Negative
FNA 4	NPC	HUSM	WT	WT	Negative
FNA 6	NPC	HUSM	MT	MT	Positive
FNA 7	NPC	HUSM	MT	MT	Positive

**Table 5:** The treatment response prediction of the InnoPrimers-duplex qPCR result of 30 bp deletion genetic biomarker against clinician treatment response prediction.

		Clinician Treatment Response Prediction			
**Treatment response prediction based on the InnoPrimers-duplex qPCR result**		**CR**	**PR**	**PD/Recurrence** *	**Total**
	**CR (Cq ≥ 37)**	15	5 ^a^	3 ^b^	23
	**PR (Cq: 36.99–32.00)**	3 ^c^	5	0	8
	**PD/recurrence** **(Cq ≤ 31.99)**	1 ^d^	0	2	3
	**Total**	19	10	5	34

Footnote: CR, the complete response was defined as disappearance of all target lesions (short axis of target or non-targeted neck pathological lymph nodes <10 mm); PR, partial response defined as a reduction of the longest diameter of target lesions by at least 30% (using the baseline sum of longest diameter as a reference); PD, progressive disease was defined as increasing the sum of target lesions’ longest diameter by at least 20% (using the smallest sum of the longest diameter since treatment began as a reference; also, one or more new lesions have emerged). * Some authors included both recurrent and progressive diseases in their description of recurrent NPC, but progressive diseases have a better result than the recurrent disease. Recurrent NPC includes local, regional or distant metastasis. ^a^ Among five patients who were detected with Cq ≥ 37 and had PR based on clinician treatment response prediction, two patients were diagnosed as WHO type II NPC, and one patient was diagnosed as WHO type I NPC. ^b^ Among three patients who were detected with Cq ≥ 37 and had PD/recurrence based on clinician treatment response prediction, two patients were diagnosed as WHO type II NPC. ^c^ Among three patients who were detected with Cq within an interval of 36.99–32.00 and had CR based on clinician treatment response prediction, two patients were diagnosed as WHO type II NPC. ^d^ One patient was detected with Cq ≤ 31.99 and had CR based on clinician treatment response prediction; this patient was diagnosed as WHO type II NPC.

**Table 6:** Association between categorical Cq values of 30 bp deletion genetic biomarker and clinician treatment response prediction in NPC WB samples (*n* = 34).

	NPC WB Sample				
		Categorical Cq of 30 bp Deletion Genetic Biomarker in WB Samples, *n* (%)			*p*-Value ^#^
		≥37	36.99–32.00	≤31.99	0.033 *
**Clinician treatment response prediction**	**CR** **PR** **Recurrence/PD**	15 (44.2) 5 (14.7) 3 (8.8)	3 (8.8) 5 (14.7) 0 (0)	1 (2.9) 0 (0) 2 (5.9)	

^#^ Fisher’s Exact Test was applied, * significant *p*-value < 0.05.

## Data Availability

The data used to support the findings of this study are available from the corresponding author upon request.

## Abbreviations

NPC = nasopharyngeal carcinoma; EBV = Epstein–Barr virus; LMP1 = latent membrane protein 1; NKC = non-keratinizing carcinoma; NKDC = non-keratinizing differentiated carcinoma; NKUC = non-keratinizing undifferentiated carcinoma; HPE = histopathological examination; WB = whole blood; NB = NPC biopsy tissue samples; AB = NPC archive biopsy sample; FNA = fine needle aspiration; CR = complete response, PR = partial response; PD = progressive disease; FDG–PET = fluorodeoxyglucose–positron emission tomography; CT = computed tomography; MRI = magnetic resonance imaging.
